# Mental Health First Aid guidelines for helping a suicidal person: a Delphi consensus study in India

**DOI:** 10.1186/1752-4458-4-4

**Published:** 2010-02-19

**Authors:** Erminia Colucci, Claire M Kelly, Harry Minas, Anthony F Jorm, Sudipto Chatterjee

**Affiliations:** 1 Centre for International Mental Health, Melbourne School of Population Health, The University of Melbourne, Parkville, Victoria 3010, Australia; 2 Mental Health First Aid Training and Research Program, ORYGEN Youth Health Research Centre, The University of Melbourne, Parkville, Victoria 3052, Australia; 3 Sangath Centre, 841/1 Alto-Porvorim, Goa 403521, India

## Abstract

**Background:**

This study aimed to develop guidelines for how a member of the Indian public should provide mental health first aid to a person who is suicidal.

**Methods:**

The guidelines were produced by developing a questionnaire containing possible first aid actions and asking an expert panel of Indian mental health clinicians to rate whether each action should be included in the guidelines. The content of the questionnaire was based on a systematic search of the relevant evidence and claims made by authors of consumer and carer guides and websites. Experts were recruited by SC, EC and HM. The panel members were asked to complete the questionnaire by web survey. Three rounds of the rating were carried and, at the end of each round, items that reached the consensus criterion were selected for inclusion in the guidelines. During the first round, panel members were also asked to suggest any additional actions that were not covered in the original questionnaire (to include items that are relevant to local cultural circumstances, values, and social norms.). Responses to the open-ended questions were used to generate new items.

**Results:**

The output from the Delphi process was a set of agreed upon action statements. The Delphi process started with 138 statements, 30 new items were written based on suggestions from panel members and, of these 168 items, 71 met the consensus criterion. These statements were used to develop the guidelines appended to this paper. Translated versions of the guidelines will be produced and used for training.

**Conclusions:**

There are a number of actions that are considered to be useful for members of the public when they encounter someone who is experiencing suicidal thoughts or engaging in suicidal behaviour. Although the guidelines are designed for members of the public, they may also be helpful to non-mental health professionals working in health and welfare settings.

## Background

As reported by WHO [1] suicide is a huge but largely preventable public health problem, causing almost half of all violent deaths and resulting in one million fatalities every year (i.e., almost 3000 every day), as well as economic costs in the billions of dollars. Estimates suggest fatalities could rise to 1.5 million by 2020. For every person who completes suicide, 20 or more make a suicide attempt, resulting in injury, hospitalization, and emotional and mental trauma, although no reliable data are available on its full extent [1,2]. Worldwide, suicide rates have increased by 60% over the last 50 years. The increase has been especially marked in developing countries [1]. The worldwide increase has been particularly alarming amongst young people aged 15 to 25 years [2].

For every suicide death there are scores of family and friends whose lives are devastated emotionally, socially and economically [2]. For these family members and friends affected by suicide or attempted suicide, the emotional impact may last for many years [1].

A member of the community who is close to the suicidal person, such as a friend, family member, co-worker or classmate, is likely to be the first person to notice hints that could be "read" as suicide warning signs by trained or experienced eyes. However, few have the knowledge and skills required to recognize the imminent risk of suicide and to assist in preventing suicide. For this reason, we decided to produce general guidelines for how a member of the public should provide first aid to a person who is suicidal, i.e. has expressed suicidal thoughts or intent, whether overt or covert, or has taken action toward making a suicide attempt. Simple and practical guidelines might help such a person to encourage a suicidal individual to seek professional help or decide against suicide. These guidelines can be applied in training courses for the public.

First aid training is widespread throughout the world, giving members of the public skills to help an injured person before medical help arrives. There are many organizations offering first aid training, but the first aid practices taught in these courses generally conform to national guidelines. While first aid training is common, it generally ignores mental health crises such as how to assist a suicidal person. Nevertheless, there have been efforts to develop training for the public that does cover these issues, such as Applied Suicide Intervention Skills Training (ASIST), [3] and Mental Health First Aid (MHFA) training [4]. Unfortunately, there is limited evidence to guide the content of such training. While randomized controlled trials provide the highest standard of evidence, it is not feasible or ethical to carry out such trials to evaluate specific suicide first aid strategies. In the absence of high quality evidence, the best option for developing guidelines is expert consensus. There are formal methods for assessing expert consensus that have been used in several areas of health research. One of the most commonly used consensus method is the *Delphi process *(see [5]). There are many variants, but all involve a group of experts making private ratings of agreement with a series of statements, feedback to the group of a statistical summary of the ratings, and then another round of rating. Delphi group members do not meet, so it is possible to do studies using mail or the Internet. The output from the process is statements for which there is substantial consensus in ratings. The Delphi method has been used in health research since the mid-70 s [6]. We have used the Delphi method to develop suicide first aid guidelines for developed English-speaking countries [7], as well as mental health first aid guidelines for non-suicidal self injury [8], panic attacks [9], psychosis [10], depression [11], and eating disorders [12]. However, we do not believe these guidelines will necessarily apply in countries with very different cultures and healthcare systems. We therefore wished to explore the possibility of developing suicide first aid guidelines for a number of Asian countries. This project was undertaken to establish whether the use of the Delphi method is a feasible approach in the development of suicide first aid consensus guidelines for Asian countries. This method was previously successfully implemented in the production of first aid guidelines for psychosis in Asia [10].

The aim of this project was to produce guidelines for use in particular Asian countries on how a member of the public should provide first aid to a person who is suicidal, i.e. has expressed suicidal thoughts or intent or has made a suicide attempt. The project did not aim to test hypotheses, rather to develop guidelines on first aid actions based on the consensus of expert clinicians. The project involved undertaking separate studies in three countries: Japan, Philippines and India. These three countries were chosen because they are Asian countries with very different cultural and religious contexts, different rates of suicide, different levels of economic development, and different levels of availability of mental health services. We expected that there would be partially different views expressed by the expert panels about appropriate guidelines for mental health first aid in relation to suicide [13-16]. The present paper presents the results of the study in India. Those in Japan and Philippines will be described in subsequent papers.

To the best of the authors' knowledge, no study of this kind has been conducted in these countries before.

## Methods

The first aid guidelines were produced using: (a) a systematic search of the relevant evidence and claims made by authors of consumer and carer guides and websites; (b) development of a questionnaire on possible first aid actions which was based on the search; (c) and the consensus of panels of clinicians from each of the countries on which first aid actions should be included in the guidelines.

### Systematic search for possible suicide first aid actions in the literature

As part of the project to develop suicide first aid guidelines for developed English-speaking countries, a systematic search for possible first aid actions was carried out. This search has involved formal professional literature listed in PubMed and PsycLit and other sources such as existing general mental health first aid manuals [4], other relevant manuals and guides on suicide prevention (e.g. Suicide Prevention Skills Training, [17]; Mental Health for Emergency Departments [18]) and relevant web sites (e.g. Samaritans). This method has been previously published for suicide first aid guidelines in developed English-speaking countries [7].

### Construction of the questionnaire

A questionnaire was constructed from a content analysis of the actions indicated in the literature. Only statements that suggested a potential first aid action (i.e. what the first aider should do) or relevant awareness statements (what the first aider should know) were included in the questionnaire. These statements were grouped based on their common themes and used by a working group to generate questionnaire items specifying what actions a first aider should take. No judgments were made by the working group about the potential usefulness of the statements. Anything was included that fitted the definition of first aid, even if contradictory to other statements.

The questionnaire developed for English-speaking countries had 114 items, each describing a potential action that a first aider could do, which could be put to the panel for rating. These items covered the following broad areas: identification of suicide risk, assessing seriousness of suicide risk, initial assistance, talking with a suicidal person, no-suicide contracts, ensuring safety, confidentiality, and passing time during the crisis. The items are shown in Additional File 1. For the Asian guidelines, we added a few other items based upon the Chief Investigator's previous work on suicide prevention in Asian countries (e.g [14,19]). Thus, the initial questionnaire contained 140 first aid action items, plus 13 questions on participants' socio-demographics, experience/training and opinions on suicide first aid. Open-ended questions to generate additional culturally specific items were also included. Given that this was an exploratory project, we used English-language questionnaires, because the cost of doing it in the experts' native languages would have been prohibitive.

### Forming panels

**Figure 1:**
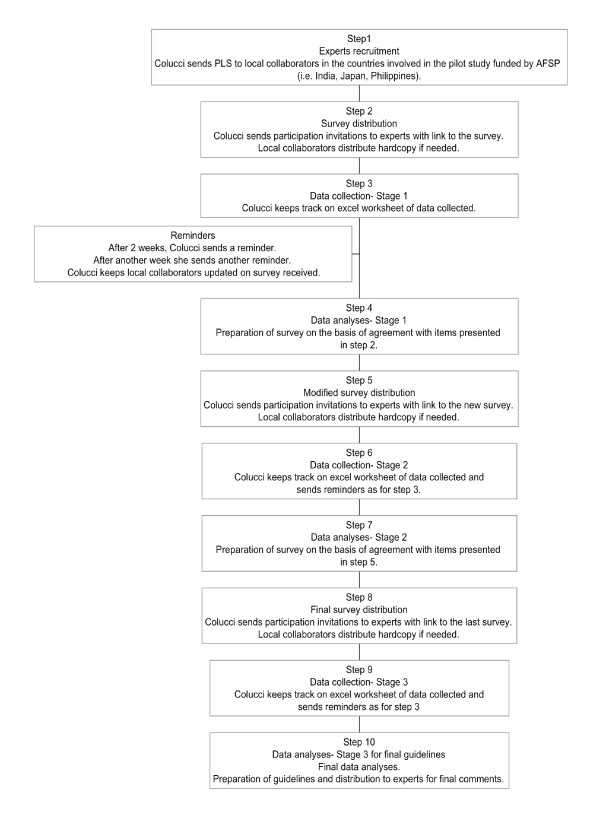
**Stages in the guidelines development**.

A panel of Indian clinical experts, currently working in India, was formed. The experts were recruited by SC, HM and EC (see Figure 1). When invitation letters, together with the Plain Language Statement, were sent to professionals asking them to be involved, they were also invited to nominate any colleagues who they felt would be appropriate panel members. During the recruitment process potential participants were informed that one of the selection criteria was to be fluent in written English. The number of panel members in previous Delphi studies has varied considerably from 15 to 60 [20]. We aimed to have a minimum of 25 members in a panel.

### Delphi process

In Round 1 of the Delphi process, panel members were asked to complete an on-line questionnaire. This was administered using the SurveyMonkey application [21], with the option to complete it by email or paper mail if this was not possible (although no participant opted for this alternative). The questionnaire consisted of a list of first aid actions to rate. Only actions that are do-able by mental health first aiders were included in the list of items to be rated. Members of the expert panel were given the following instructions to guide their judgments:

"The following questionnaire asks about the best way a member of the public can help someone who is thinking about, or planning to suicide. Mental health first aid is defined as help given to someone who is experiencing a mental health problem, or is in a mental health-related crisis, until professional help is received or the crisis resolves. It does not include counselling or therapy. In the case of suicide, mental health first aid is given until the person decides to accept professional help, or decides against suicide. People who offer mental health first aid may be friends, family members, colleagues or acquaintances. They may or may not be involved in the person's life before or after offering first aid. For brevity, we will refer to the person offering assistance as "the first aider". When completing this questionnaire, you will read statements describing possible actions that the first aider can take to assist a suicidal individual. You will be asked to rate how important each item is as a guideline for a first aider. Please rate as "essential" or "important" those items which you feel should guide most people, most of the time, when assisting a suicidal person. The statements in this questionnaire were derived from a search of both professional and lay literature in English-speaking western countries. Therefore, there will be actions which would be appropriate for members of the public in your country which are not included and there will be actions that may be appropriate in Western countries but not in your cultural context. At the bottom of each page, there is room for you to add suggestions. Please consider the cultural, social and religious environment where you live, and try to add some relevant suggestions on each page. The more panel members add to this questionnaire, the more relevant and useful the guidelines will be for each individual country. Thank you for taking the time to assist us in this important suicide prevention project!".

The definition of mental health first aid given to the panel was: "Mental Health First Aid is the help provided to a person developing a mental health problem or in a mental health crisis. The first aid is given until appropriate professional treatment is received or until the crisis resolves." This definition distinguishes a first aider's role from that of a clinician. In the case of a suicidal person, the first aider responds by getting professional help for the person, and supporting the person and ensuring their safety until the crisis has passed. The guidelines needed to focus on the immediate prevention of suicide, and not on solving the problems that lead to the crisis.

Panelist members were asked to rate each statement according to how important they believed it was as a potential first aid guideline for helping a suicidal person. The response scale was: 1. Essential; 2. Important; 3. Don't know/Depends; 4. Unimportant; 5. Should not be included. The scale was purposefully asymmetric because only items with positive ratings were of interest for the guidelines. This scale has worked well in previous guideline development work [7].

At the end of each block of items, the panel members were asked to give any comments or add any additional actions that were not included in the questionnaire. The suggestions made by the panel members in response to the open-ended questions were reviewed by the research team and used to construct new items. Suggestions were accepted and added to Round 2 if they represented a truly new idea, could be interpreted unambiguously and were actions. Suggestions were rejected if they were near-duplicates of items in the questionnaire, if they were too specific, too general or were more appropriate to therapy than first aid. This was the place where culturally specific material could be introduced.

Responses were analysed to give the percentage of the panel who rated an item as either "essential" or "important". Items for which there was at least 80% consensus were included in the guidelines. Items were re-rated if 70-79% of the panel rated them as "Essential" or "Important". A small number of items that received more than 50% of "Don't know/Depends" or "Not sure" answers were reworded, to make them clearer, and re-rated. For example, 'contact the person's spiritual or religious leader' became 'contact the person's spiritual or religious leader, if they have one. 'Items that met neither condition were rejected.

In Round 2 a second questionnaire was prepared. This consisted of any new item that was generated from the comments in Round 1, plus items that were positively rated by at least 70% of respondents but did not reach the 80% criterion for inclusion. Participants received an email with an individualized link to the online survey and a word file that, together with the latter items, fed back a statistical summary of the items that were to be re-rated (i.e. their own original response to the item together with total percentages of endorsement of the item). They were told that they did not have to change their responses when re-rating an item but that, if they wished, they would have the opportunity to do so. Panel members were asked to re-rate items which approached the desired level of consensus and rate the new, culturally specific items after reading this feedback letter. At the end of this round, any item that reached the 80% consensus criterion was selected for inclusion in the guidelines.

In Round 3, any of the new items generated from the open-ended questions in Round 1 that did not reach consensus in Round 2 went back for re-rating (together with a few items which received an excessively high number -more than 50%- of "Don't know/depends" answers). Participants received a statistical summary of the results as for Round 2. Again, items that reached the required level of consensus were included in the guidelines.

### Cultural appropriateness of guidelines

As described above, the questionnaire on potential first aid actions that was developed for English-speaking countries (with a few additional/modified items) was the starting point for the Round 1 of the Delphi process for this project. At each round, panel members were asked to suggest additional actions that were not covered in the original questionnaire, specifically to include items that were relevant to local cultural circumstances, values, etc. These new items were rated in Rounds 2 and 3. Panel members were also invited to comment on items that were in the initial questionnaire that they considered to be culturally irrelevant or unacceptable, or that would not be feasible because of the local health system and other resources. Moreover, in the formation of the expert panels in each country, we were careful to include as wide a representation (cultural and geographic) of professionals as possible.

### Ethics

Ethics approval was obtained from the University of Melbourne Human Research Ethics Committee (Project No. HREC 0605537).

## Results

### Sample

In India, 30 panel members were involved in Round 1 (i.e. 68% of the experts who were invited to participate), 25 in Round 2 and 23 in Round 3. All panel members were currently working in India. The majority were psychiatrists (63%) and psychologists (27%). Two participants were social workers and one was an occupational therapist. The panel comprised of 17 males and 13 females. The majority (57%) of the participants were in the age range 40-49 years, 3 were aged 18-29 years, 6 aged 30-39 years, and 4 aged 50-59 years.

Some information was also collected on the clinical experience of the panel members. On average participants reported that they had practiced in mental health/psychiatry for 15 years (the shortest time was 2 years and the longest 30 years). Less of a quarter of the participants (23%) received some formal education related to their profession overseas (mainly in UK and one in Australia). Slightly over a third of the participants (37%) reported having received a formal training specifically on suicide prevention/intervention. However, when asked to state how well prepared they felt to assist a suicidal person, 3.3% answered "Not at all", 23% "somewhat prepared", 47% "mostly prepared" and 27% "very prepared". Although participants generally felt prepared to assist a suicidal person, in their opinion most people in India are not at all prepared (53%) or somewhat prepared (43%). Only one person believed others are mostly prepared to assist.

### Items endorsement

After three Delphi rounds, there were 71 items that were rated as "essential" or "important" by 80% or more of the panel members.

At Round 2, 30 new items suggested by participants were added to the questionnaire. The followings are examples of such items:

• An important warning sign for suicide is if a person is saying they wish or intend to see or speak to someone who is dead (e.g., a deceased family member).

• An important warning sign for suicide is if a person is expressing in words or actions a sense of shame (e.g. from failure or loss).

• The first aider should not offer false hope, or make unrealistic promises.

• The first aider should not dismiss the person's feelings or compare their problems to the problems of others.

• When talking to the suicidal person, the first aider should use the person's belief systems and values to encourage them to change their mind about suicide.

• The first aider should contact the elders in the person's community.

A number of responses to the Round 1 open-ended questions did not meet criteria for creation of a new item (e.g. they did not fit the definition of first aid or did not suggest a clear action) or were comments/suggestions. The following are examples of the comments and suggestions that did not generate new items:

• " The idea of suicide first aid does not exist in our country. It should be strongly encouraged at all levels, especially in schools as the prevalence of suicide among school kids is alarming (...)".

• " I think that there should be more awareness about "suicide" in our country so that people are sensitized about the "warning signs" of this person even before he can attempt suicide. In my culture, since the family is such an important support system, they must be informed right from the start".

• " Psychiatrists, psychologists and other mental health professionals should receive training in how to handle suicidal individuals, victims of attempted suicide, and survivors of suicide".

• "In our society individual freedom and opinion is not as important as collective opinion. Hence it is not difficult to dissuade suicide by involving other individuals in the family and society. However, the motivation of "others" is not always high".

• "In eastern culture, people are less likely to take the issue seriously when one discloses one's intention to commit suicide".

See the Additional file 1 for a complete list of rated statements, including the percentage of panel members endorsing each item.

At the end of the survey, participants were asked their opinions about the likely effectiveness of suicide first aid, using a 5-point Likert scale (from "definitely yes" to "definitely no"). All of them believed that if the first aider does the right thing the risk of suicide can be reduced. Substantial proportions of the respondents thought that if the first aider does the wrong thing the risk of suicide can definitely (27%) or probably (45%) be increased (21% answered "Don't know/depends" and 14% "Probably no").

The longer-term goal of the project is to use the guidelines to develop, implement and evaluate a training program on suicide first aid in India. When asked if they thought members of the public should receive such training, 69% of panel members responded "definitely yes" and 27% "probably yes". Only one respondent answered "don't know/depends".

### Development of statements that constitute the guidelines

The output from the Delphi process was a set of agreed upon action statements. The statements refer to actions that can be done by a mental health first aider. To be usefully communicated, these action statements have been woven into an integrated piece of text (instead of a list of "dos" and "don'ts"), which become the Suicide First Aid Guidelines (see Additional File 2).

### Participant feedback and modification of draft guidelines

The draft guidelines were sent to all panel members for their comments and final endorsement. Since the guidelines were meant to be useful to members of the public, it was important to ensure that they were written to be comprehensible to the target non-professional readership. Feedback from panel members was explicitly sought on the structure and readability of the guidelines, and suggested improvements were incorporated in the final version.

## Discussion

This project has demonstrated that it is possible to achieve consensus among mental health professionals on first aid strategies for suicidal thoughts and behaviour, and that the Delphi method is suitable for developing consensus guidelines in India. The method has been similarly successful in Japan and the Philippines. We would suggest that guideline development studies, using a similar method, could be carried in a number of other countries. As well as developing country specific guidelines, it will also be possible to develop guidelines that are appropriate for cultural minorities within a country. This approach has been used in Australia, with a separate Delphi study undertaken to develop guidelines for Aboriginal Australians, using Aboriginal mental health experts as panel members [22], and specific teaching programs have been developed for non-English speaking immigrant communities [23].

The next steps will be dissemination and use of the guidelines for the purpose of increasing community members' ability to recognize the risk of suicide and undertake basic first aid actions. A number of panel members, in collaboration with our research team, are currently seeking further funding so that the guidelines can be translated into local languages, and to examine whether modifications are required for cultural minority groups. The guidelines that have been developed will serve as a basis for detailed work on culturally appropriate guideline development in languages other than English and for specific national and sub-national cultural groups.

These guidelines can be used as a source of advice to the public, as a basis for determining the curriculum of first aid training courses, and as a standard against which to evaluate the quality of existing materials and programs. The guidelines will inform the development of culturally appropriate training programs and information materials for how a member of the public can assist someone who is suicidal. In some countries and areas with less developed healthcare systems, we believe the guidelines will be useful for primary health workers as well as for members of the public. It is anticipated that the results of this project will contribute to a program of training for community nurses and midwives and for village mental health workers to enable them to contribute more effectively to suicide prevention programs. It will of course be necessary to rigorously evaluate the impact of such training programs [23-25].

### Limitations

One limitation of this study is the small number of panel members, although Delphi studies have been successfully run even with smaller groups.

We have developed these guidelines for India as a whole, while recognizing that India is characterised by remarkable cultural and linguistic diversity. It is possible that the guidelines are not applicable to minority cultures within India.

The questionnaire was administered in English rather than in the panelists' native languages. This of course limits the general applicability of the findings. It is possible that there would have been more culturally specific responses if panelists had used their native language.

Future studies should recruit broader and more representative expert panels including, where possible, professionals from all the relevant mental health disciplines and consumer and carer representatives. This is presently difficult in many Asian countries because there are very few (if any) clinical psychologists, psychiatric social workers, occupational therapists and mental health nurses, and the participation of consumers and carers in such research, as members of an 'expert panel' rather than as research subjects, is still uncommon [10].

Another limitation is that the inclusion of culturally relevant material was dependent on panelists responding to the open-ended questions and not every participant did this. This may have been, in some cases, due to lack of time or because the participants in this study were better able to read English than to write it. It may also be because, in questionnaires of all kinds that require ratings to be made, respondents rarely take the opportunity to write comments or to make suggestions when the opportunity is given [10]. However, it must be noted that, compared to other similar mental health first aid guideline research, a considerable number of suggestions for new items were given. This might have been because of the emphasis in the instructions to participants on providing suggestions based on participants' cultural, social and religious settings in each section of the questionnaire.

## Conclusions

There is a growing awareness of suicide as a major public health problem, even though there is a taboo in many societies against discussing it openly [1]. Developing suicide first aid guidelines for community members, and training programs based on these, might also contribute towards changing society's attitudes towards suicide and people who consider suicide.

This study has demonstrated that it is possible to reach consensus for the development of guidelines for India. Although the guidelines were designed for the public, they may also contain advice that might be helpful to people working in health and welfare professions.

Where the guidelines are used as the basis for first aid training, efforts need to be made as far as possible to evaluate their impact on the first aider's helping behaviour and on the recipients of the first aid. This will assist researchers to develop an evidence base for mental health first aid and suicide prevention initiatives.

In a WHO news release [2] it was stated that "It's important to realise that suicide is preventable". By collaborating with local experts to agree on a minimum set of suicide first aid actions, and by making such guidelines freely and easily accessible to everyone, we hope to convey the message that suicide is preventable, suicide is everyone's business, and everyone can contribute to its reduction. Members of the general public have a crucial role to play in suicide prevention. Creating opportunities for the public to learn basic first aid actions, and how to implement them when needed, is an important step towards more effective suicide prevention.

## Competing interests

The authors declare that they have no competing interests.

## Authors' contributions

EC recruited clinical experts, revised the existing questionnaire, prepared and administered the on-line surveys for all three rounds, analysed the data, prepared the draft and final guidelines and wrote the first draft of the manuscript. CMK wrote the former questionnaire and contributed to the revised version, supervised every stage of the project and co-wrote the guidelines. HM co-wrote the grant application, recruited the main local collaborators and clinical experts, reviewed the Round 1 questionnaire and the guidelines. AFJ co-wrote the grant application and developed the method. SC was the main local collaborator in the study and recruited the majority of the clinical experts. All authors contributed to the writing of the manuscript and approved the final version.

## Supplementary Material

Additional file 1 Table of data showing the items included in the Delphi survey and the endorsement levels from the Indian panel members. Click here for file

Additional file 2 **First aid guidelines for India**. This file may be distributed freely, with the authorship and copyright details intact. Please do not alter the text or remove the authorship and copyright details. Click here for file
